# Using routine outcome measures as clinical process tools: Maximising the therapeutic yield in the IAPT programme when working remotely

**DOI:** 10.1111/papt.12400

**Published:** 2022-05-16

**Authors:** Cintia L. Faija, Penny Bee, Karina Lovell, Nicky Lidbetter, Judith Gellatly, Kerry Ardern, Kelly Rushton, Helen Brooks, Dean McMillan, Christopher J. Armitage, Rebecca Woodhouse, Michael Barkham

**Affiliations:** ^1^ School of Health Sciences, Division of Nursing, Midwifery and Social Work, Manchester Academic Health Science Centre University of Manchester Manchester UK; ^2^ Greater Manchester Mental Health NHS Foundation Trust Manchester UK; ^3^ Anxiety UK Manchester UK; ^4^ The Big Life Group, UK Manchester UK; ^5^ Clinical and Applied Psychology Unit, Department of Psychology University of Sheffield Sheffield UK; ^6^ Hull York Medical School and Department of Health Sciences University of York York UK; ^7^ Manchester University NHS Foundation Trust Manchester Academic Health Science Manchester UK; ^8^ Centre NIHR Greater Manchester Patient Safety Translational Research Centre Manchester UK; ^9^ Manchester Centre for Health Psychology University of Manchester Manchester UK

**Keywords:** clinical feedback, collaborative conversational approach, depression, IAPT, mental health, psychological practitioner, qualitative study, routine outcome measures, step 2, telephone treatment, wellbeing, wellbeing anxiety

## Abstract

**Objectives:**

The objective of the study was to investigate the administration and use of routine outcome monitoring session by session in the context of improving guided‐self‐help interventions when delivered remotely at Step 2 care in the English Improving Access to Psychological Therapies (IAPT) services.

**Design:**

Qualitative research using recordings of telephone‐treatment sessions.

**Method:**

Participants (11 patients and 11 practitioners) were recruited from four nationally funded IAPT services and one‐third sector organisation commissioned to deliver Step 2 IAPT services, in England. Data collection took place prior to the COVID‐19 pandemic. Transcripts of telephone–treatment sessions were analysed using thematic analysis.

**Results:**

Four themes were identified: (1) lack of consistency in the administration of outcome measures (e.g. inconsistent wording); (2) outcome measures administered as a stand‐alone inflexible task (e.g. mechanical administration); (3) outcome measures as impersonal numbers (e.g. summarising, categorising and comparing total scores); and (4) missed opportunities to use outcome measures therapeutically (e.g. lack of therapeutic use of item and total scores).

**Conclusions:**

The administration of outcome measures needs to ensure validity and reliability. Therapeutic yield from session‐by‐session outcome measures could be enhanced by focusing on three main areas: (1) adopting a collaborative conversational approach, (2) maximising the use of total and items scores and (3) integrating outcome measures with in‐session treatment decisions. **S**hifting the perception of outcome measures as impersonal numbers to being process clinical tools ensures a personalised delivery of psychological interventions and has the potential to enhance engagement from practitioners and patients what may reduce drop‐out rates and improve clinical outcomes.

**Practitioner points**

- There is room to improve low‐intensity psychological interventions delivered by telephone by optimising the use of session‐by‐session outcome measures. Moving towards a collaboratively conversational approach of session‐by‐session measurement establishes outcome measurement as a clinical process tool embedded in treatment leading to a personalised delivery of psychological interventions.
- We hypothesise that therapeutic use of session‐by‐session measures would enable services and practitioners to better utilise standard procedures to inform psychological interventions.
- We anticipate shifting the perception of outcome measures as impersonal numbers to being process clinical tools as having the potential to enhance engagement from practitioners and patients that may potentially reduce early attrition rates and influence clinical outcomes.
- We would recommend placing more emphasis/dedicated time to the ability to use outcome measures from a collaborative conversational approach; making sure this is within the low‐intensity competencies, assessed during training and monitored in clinical supervision.

## BACKGROUND

The UK policy and guidelines recommend the use of routine outcome monitoring to ensure psychological therapies are delivered effectively and efficiently to the best standard of care (Department of Health, 2012). These initiatives underpin the English Improving Access to Psychological Therapies (IAPT) programme—a stepped care model—launched nationally in 2008, which aims to provide evidence‐based psychological therapies for people with common mental health conditions (e.g. anxiety and depression), following National Institute for Health and Care Excellence (NICE) guidelines.

Step 2 care in IAPT services involves low‐intensity cognitive behavioural therapy, which is guided‐self‐help supported by didactic materials delivered in a variety of formats (e.g. face to face, telephone, group) over a maximum of eight sessions. Step 2 care is delivered by psychological well‐being practitioners (PWPs) trained over one year to a standardised curriculum accredited by the British Psychological Society.

IAPT heralded a change in direction for mental health services and providers with an emphasis placed on the routine collection of sessional outcome data. Sessional outcome data informed the status of treatment in relation to discharge (improved) at Step 2 or stepping up to more intense interventions at Step 3 and marked a move away from services relying either on clinical judgement or paired pre/post‐treatment data when it was available. In contrast, IAPT has recorded 98% outcome data completeness for pre/post‐treatment scores from all clinical contacts (IAPT, 2021).

The move from low rates of pre–post measurement to high rates of session‐by‐session measurement has changed the assessment of patient progress in psychological treatments. The IAPT manual highlights how the use of session‐by‐session outcome measures aims to benefit services, practitioners and patients (IAPT, 2021). However, qualitative research has identified some key challenges that warrant discussion to further understand how to maximise their potential use in routine clinical practice during treatment.

At a service level, outcome measures can be used to monitor and support service performance, address variability across practitioners and enhance the overall quality and cost‐effectiveness of service delivery (IAPT, 2021). However, research focussing on local and national decision makers shows that the completion of outcome measures session by session eroded valuable clinical time and presented a potential social desirability risk, influencing patients to report improvements due to the on‐the‐spot nature of being questioned about their symptoms by telephone (Rushton et al., 2019).

At a practitioner level, session‐by‐session outcome measures can provide information to identify targets for interventions and for session work, help to inform the appropriateness of treatment, evaluate treatment effectiveness, support a discussion of clinical cases in supervision and facilitate conversations on difficult topics (IAPT, 2021). Research on practitioners' attitudes, though suggest that perceived additional burden on patient, time allocation, adequacy of outcome measures, fear of interfering with the therapeutic alliance and therapeutic process, anxieties about performance monitoring, and lack of awareness of the benefits for its use, may limit engagement with outcome measures (Rao et al., 2010).

At a patient level, session‐by‐session outcome measures have the potential for people to see their progress and improvements over time, contribute to the understanding of their problem, enhance engagement and support the development of the therapeutic relationship (IAPT, 2021). Patients can find the visualisation of the changes in a graph useful, and the discussion of scores in the session with their therapist helpful in increasing self‐awareness (Unsworth et al., 2011). However, research on patients' attitudes to receiving psychological treatment by telephone in IAPT has identified that completing measures can be perceived as anxiety‐provoking, time‐intensive, business‐like, scripted and impersonal (Rushton et al., 2020). These findings are consistent with an analysis of practitioner–patient interactions exploring audio recordings of telephone assessments conducted in IAPT services (Drew et al., 2021).

Despite the benefits of session‐by‐session use of outcome measures, challenges faced in clinical practice remain unresolved. Evidence on the value of providing feedback and facilitating a patient–practitioner dialogue about outcome measure monitoring is emerging and could prove fruitful in maximising their use during ongoing treatment (Barkham et al., 2015; Cross et al., 2015; Lambert et al., 2018; Unsworth et al., 2011). For example, Cross et al. (2015) reported on the clinical relevance of focusing on changes at the level of individual questions rather than the total score level, thereby facilitating a collaborative dialogue based on individual‐item scores.

The COVID‐19 pandemic has forced the adoption of remote delivery methods that may not have been so widely used pre‐pandemic, including telephone and online video, meaning there is a gap in knowledge on the use of session‐by‐session measurement when psychological interventions are delivered remotely. In light of this context, the current study aimed to explore the administration and therapeutic use of routine outcome monitoring during treatment delivered by telephone at Step 2 care in IAPT services.

## METHOD

### Design

The study used qualitative methods to analyse digital recordings of patient–practitioner interactions of telephone‐delivered treatment sessions in IAPT services. The study adopted a social constructionist approach/paradigm, viewing knowledge, meaning and experience as socially constructed rather than residing purely within individuals (Burr, 1995; Gergen, 1985). The study forms part of a larger programme of work to enhance the quality of psychological interventions delivered by telephone at Step 2 care in IAPT services (i.e. EQUITy). Ethical approval for the EQUITy Research Programme was granted by North West Greater Manchester West Research Ethics Committee (Ref: 18/NW/0372). Governance was approved at all participating IAPT services (NHS and third sector).

### Recruitment and data collection

Patients and PWPs were recruited from five IAPT services across the North and East of England, four were nationally funded, and one was a commissioned provider within the charitable sector. PWPs were eligible if they were delivering interventions by telephone. Patients were adults (18 years of age or older) with common mental health problems (anxiety, depression) receiving Step 2 care in IAPT services delivered by telephone.

Data collection took place between September 2018 and July 2019 (i.e. prior to the COVID‐19 pandemic). Data were collected from 18 PWPs and 106 patients, comprising 123 recordings. All participants provided consent to take part and to be recorded. From the 123 recordings, 66 were assessments and 57 were treatment sessions (37 Session 1 and 20 Session 2). This study focused on treatment sessions to explore the implementation and therapeutic use of outcome measures, meaning a subsample of the full data set was used. For findings related to assessments, please see Irvine et al. (2020) and Drew et al. (2021).

### Study data set

The complete treatment data set included 57 transcripts corresponding to 11 practitioners and 40 patients. Six of the 11 practitioners provided multiple recordings (*n* = 52). To allow exploration of the use of outcome measures across practitioners and between sessions within the same dyad (patient–practitioner), a subsample of 16 transcripts was selected. The subsample was selected to include one transcript for each of the 11 practitioners that provided recordings of treatment sessions, and five transcripts that corresponded to the same patient–practitioner dyad. For practitioners providing multiple recordings, transcripts were randomly selected.

A subsample of 16 transcripts were analysed, nine were Session 1 and seven were Session 2. The average duration for Session 1 was 34 min (Range 27–42 min) and 30 min for Session 2 (Range 27–37 min). The recordings were transcribed verbatim by an independent transcription company (approved by the University of Manchester), and transcripts were checked for accuracy by two researchers. Following transcription, any identifiable information was removed from the transcripts in accordance with ethics guidelines. Data were securely stored in online servers at the Universities of Manchester and York.

### Participants

Nine of the 11 practitioners were women and the mean age was 35 years (*SD* = 13.0, range 26–72). The average length of experience delivering psychological interventions was 3 years (*SD* = 2.4 years, range 0.5–7.5 years).

From the 11 patients included in the subsample, ten were women and the average age was 38 years (*SD* = 14.0, range 23–53). Six were diagnosed with anxiety, one with depression and four with both (anxiety and depression); four patients reported co‐morbid physical health conditions, and four were employed.

### Routine outcome measures

Outcome measures used in this sample comprised four questionnaires: the Patient Health Questionnaire (PHQ‐9; Kroenke et al., 2001); the Generalised Anxiety Disorder Scale (GAD‐7; Spitzer et al., 2006), the IAPT phobia scale (IAPT Toolkit, 2008) and the Work and Social Adjustment Scale (WSAS; Mundt et al., 2002). Table 1 includes a description of each of the measures.

**TABLE 1:** Description of routine outcome measures

Patient health Questionnaire‐9 (PHQ‐9; Kroenke et al., 2001)	The PHQ‐9 is a 9‐item measure of the severity of depression using a two‐week timeframe with items rated on a 4‐point scale from ‘not at all’ (0) to ‘nearly every day’ (3). Total scores of 5, 10, 15, and 20 represent cut‐points for mild, moderate, moderately severe and severe depression, respectively
Generalised Anxiety Disorder‐7 (GAD‐7; Spitzer et al., 2006)	The GAD‐7 is a 7‐item measure of the severity of anxiety rated over the past two weeks. Responses are on a 4‐point scale from ‘Not at all’ (0) to ‘Nearly every day’ (3). Total scores of 5, 10 and 15 are taken as cut‐off points for mild, moderate and severe anxiety respectively
Work and Social Adjustment Scale (WSAS; Mundt et al., 2002)	The WSAS assesses the extent to which a person's mental health problem interferes with their functioning at work, home management, social and private leisure activities and with family/relationships
IAPT phobia scale (IAPT toolkit, 2008)	The IAPT phobia scale is a 3‐item measure assessing avoidance due to fear of outlined situations (panic attacks, social situations or specific situations such as driving, flying, heights and blood). Items are rated depending on how much the patient avoids the circumstances described on a scale from 0 (‘Would not avoid it) to 8 (‘Always avoid it’). A total score of 8 or above would indicate symptoms of panic disorder

### Data analysis

Data were analysed using thematic analysis at a semantic level (Braun & Clarke, 2006) to identify and report on patterns related to the administration and clinical use of outcome measures during treatment sessions. NVivo Pro (QSR International, 2018, version 12) was used to manage the data during the six‐step process of analysis as described by Braun and Clarke (2006): (1) familiarising with the data; (2) generating initial codes; (3) searching for themes; (4) reviewing themes; (5) defining and naming themes; and (6) producing the report. To facilitate familiarisation with the data, all transcripts were read in full by the first author and then attention was focused on sections where practitioners administered the outcome measures and where information related to these was provided. Following familiarisation, relevant sections of the transcripts were coded inductively generating initial codes by the first author. Subsequently, initial codes were grouped into sub‐themes, which were then clustered into four main themes. Sub‐themes and themes were regularly discussed and reviewed by the wider team, and further defined and named during the writing up of the manuscript.

### Quality

The study was conducted in line with the Consolidated Criteria for Reporting Qualitative Research (COREQ) checklist (Tong et al., 2007) and followed accepted guidelines to ensure quality, validity and reliability in qualitative research (Elliot et al., 1999; Yardley, 2000).

### Team and reflexivity

CF led the data analysis and has a PhD in Psychology and clinical experience delivering different evidence‐based treatments. All members of the team had experience in qualitative research and seven had clinical experience (within or outside IAPT services; CF, MB, KL, KA, DM and NL). In addition, MB, KL and DM have experience in training practitioners; and NL is a strategic mental health lead with experience of managing IAPT services. All authors have a shared perception about the value of outcome measures in treatment effectiveness and service delivery.

## RESULTS

The analysis yielded four themes; the first two related to the *administration* of outcome measures and were labelled ‘Lack of consistency in the administration of the outcome measures’ and ‘Outcome measures administered as a stand‐alone inflexible task’. The other two themes focused on information about the *lack of therapeutic use of outcome measures* within and between treatment sessions and were labelled ‘Outcome measures as impersonal numbers’ and ‘Missed opportunities to use outcome measures therapeutically’. Table 2 provides information on the research questions together with the four themes and their corresponding sub‐themes.

**TABLE 2:** Research questions, themes and sub‐themes

Research questions	Theme	Sub‐themes
How are standardised outcome measures administered when they are completed session by session over the telephone?	(1) Lack of consistency in the administration of outcome measures	(1) Inconsistent wording to present questionnaire items and rating options
		(2) Conflicting information
		(3) Inconsistencies in the rationale for using outcome measures
	(2) Outcome measures administered as a stand‐alone inflexible task	(1) Stand‐alone expedited task
		(2) Mechanical administration
		(3) Engaging in conversation after the administration of the outcome measures
What is the therapeutic in‐session and between‐session use of outcome measures during treatment when interventions are delivered by telephone?	(1) Outcome measures as impersonal numbers	(1) Summarising, categorising and comparing total scores
		(2) Using closed questions vs. open questions
	(2) Missed opportunities to use outcome measures therapeutically	(1) Lack of therapeutic use of item scores
		(2) Lack of therapeutic use of the Phobia Scale and the WSAS

### Theme 1: Lack of consistency in the administration of outcome measures

Differences across and within practitioners were evident with inconsistent use of the formal and standardised wording of the items presented in the questionnaires, differences in the rationale for its use, lack of clarity when presenting the rating options and conflicting information provided during the session. The theme comprised three sub‐themes (see Table 2).

#### Sub‐theme 1: Inconsistent wording to present questionnaire items and rating options

Often, items were shortened or rephrased. For example, item 6 on the PHQ‐9 reads ‘Feeling bad about yourself ‐or that you are a failure or have let yourself or your family down’, and the practitioner shortened this to ‘feeling bad about yourself’ (Patient ID 35‐Practitioner ID 19‐Session 1). Item 8 on the PHQ‐9 reads ‘Moving or speaking so slowly that other people could have noticed? Or the opposite ‐being so fidgety or restless that you have been moving around a lot more than usual’ and this was rephrased as ‘Any changes to your movements? Have you noticed you moving or speaking more slowly or the opposite, that you've been more fidgety or restless?’ (Patient ID 100‐ Practitioner ID 03‐ Session 2).

Similarly, data showed a lack of consistency in the presentation of the rating options. Rating options were presented using either the categorical (e.g. not at all) or the numerical options (e.g. 0); both options were rarely presented. On multiple occasions, patients provided an answer outside of the rating options, and there was usually no attempt to clarify this. For instance, patients replied using words such as *‘all the time’*, *‘definitely’*, *‘half the time’*, making their answers ambiguous where the options were *‘Nearly every day (3)’*, *‘More than half the days (2)’*, *‘Several days (1)’*, *‘Not at all (0)’*. In a minority of cases, some practitioners attempted to gather clarification:

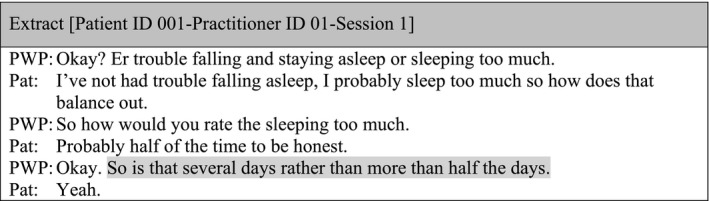

There were occasions where rating options presented by the practitioner were differ**e**nt from those stated in the questionnaires (e.g. ‘So, your options are [when referring to the PHQ‐9] “not at all, some days, most days, or every day” (Patient ID 035‐Practitioner ID 19‐Session 1) and rating options were usually only introduced at the beginning of the questionnaire.

#### Sub‐theme 2: Conflicting information

There were data showing the PWP providing further explanation of an item when there was a misunderstanding by the patient, but after the clarification, there was no review of scores. The following extract illustrates a dialogue between patient‐practitioner related to item 3 of the IAPT Phobia Scale: ‘How much do you avoid certain situations because of a fear of particular objects or activities (such as animals, heights, seeing blood, being in confined spaces, driving or flying)’:

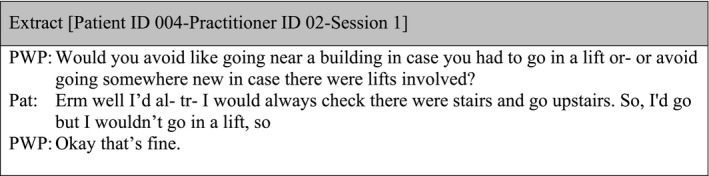

Similarly, there were times when ratings provided during the administration of the measures were not consistent with the qualitative narrative given by the patient later on in the treatment session, and these inconsistencies were not explored and ratings remained unchanged.

#### Sub‐theme 3: Inconsistencies in the rationale for using outcome measures

Data analysis revealed a lack of consistency in the rationale for the use of outcome measures session by session when presented to the patients. Data from Session 1 often indicated that outcome measures were presented to patients as something that needs to be done at every treatment session without providing a rationale.

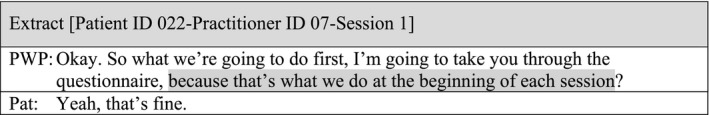

In a few recordings, practitioners provided a rationale, which included collecting information about symptoms, monitoring progress, checking treatment is on track and facilitating a conversation about changes over time; an extract illustrating this is included below:

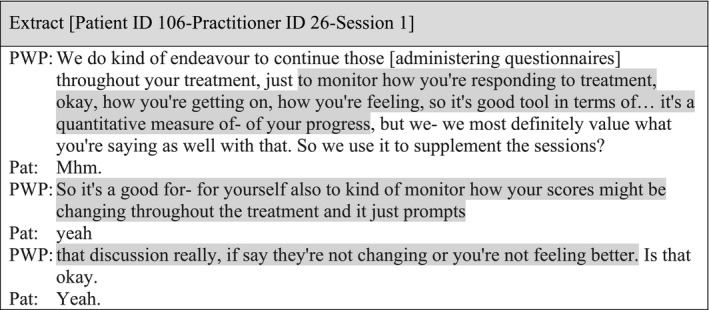

### Theme 2: Outcome measures administered as a stand‐alone task lacking on flexibility

Consistencies were identified in the implementation of outcome measures, highlighting this was done as a stand‐alone, expedited, mechanical exercise not integrated within the treatment session; patients were invited to expand on their experiences immediately after completing the measures. This theme comprised three sub‐themes (see Table 2).

#### Sub‐theme 1: Stand‐alone expedited task

Outcome measures were always administered at the beginning of treatment sessions as a stand‐alone exercise, disconnected from what happened later on in the session. Measures were positioned as an encumbrance rather than an opportunity to understand a patient's experiences. Patients were usually asked to complete the measures before the session if possible as it ‘saves a bit of time’ (Patient ID 001‐Practitioner ID 01‐Session 2), and when they were completed in the session, its completion was usually rushed. This expedited approach in the administration of measures was evident in many of the patient–practitioner interactions. An extract illustrating this is included below:

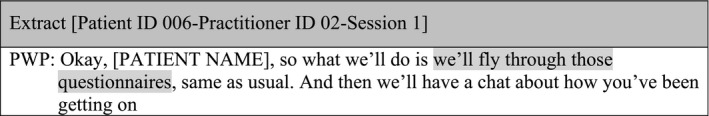

#### Sub‐theme 2: Mechanical administration

Patients sometimes provided a narrative description of their experiences or circumstances to an item question and this information was omitted, forgotten or disregarded by practitioners. Hence, although outcome measures were eliciting additional information, on multiple occasions the focus was on identifying a numerical or categorical response and closing down a patient's story.

The administration of the measures was often mechanical and lacking flexibility. The extract below illustrates how the patient provided details in relation to a sleeping question elicited in the PHQ‐9, but the practitioner avoided this information and moved on to the next item.

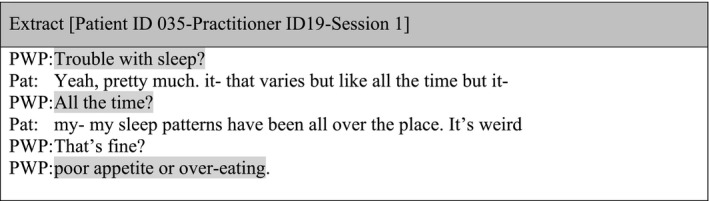

However, while patients' extended accounts were generally avoided, responses to suicide risk were picked up subsequently, although not immediately:

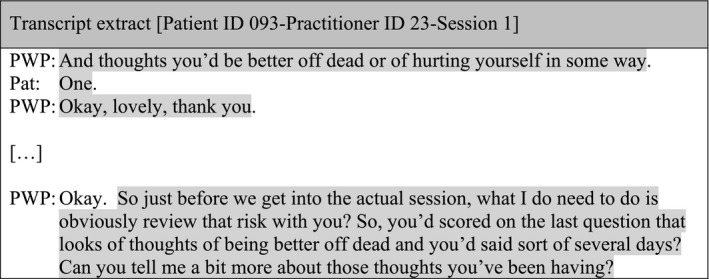

#### Sub‐theme 3: Engaging in conversation outside the administration of outcome measures

After the administration of the outcome measures, PWPs often provided a new opening to the session inviting patients to talk about their personal experiences and feelings since the last session. This demonstrated the value placed on a patient's story but was disconnected from the administration of the measures:

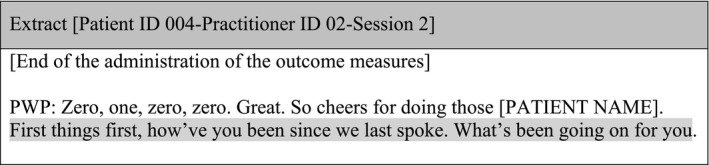

### Theme 3: Outcome measures as impersonal numbers

When patients completed the outcome measures in advance, PWPs usually did not use the scores, and when they were completed within the session, the use of this information was limited to summation, categorisation and/or comparison of total scores. In addition, this theme captured a difference between using closed and open questions after providing feedback to patients on their total scores. This theme comprised two sub‐themes (see Table 2).

#### Sub‐theme 1: Summarising, categorising and comparing total scores

Data from the administration of outcome measures during treatment sessions revealed total scores were not always produced; when they were, this applied to PHQ‐9 and GAD‐7, and the use of this information was limited to providing a numerical feedback, categorising illness severity (i.e. low/moderate/severe) and/or comparing total scores between sessions. Usually, the practitioner provided this information to patients (e.g. reduction, maintenance or increase on total score) and patients were generally passively receiving it; afterwards, the session moved quickly to address a different topic:

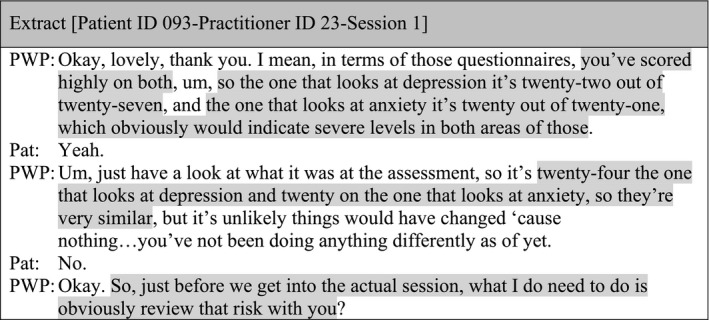

Patients often provided information about an overall feeling of improvement or deterioration, but this was not crosschecked or linked to total scores.

#### Sub‐theme 2: Using closed questions vs. open questions

There were only four examples in the data where practitioners were proactive in looking for explanations behind the total scores, which were indicating a reduction, an increase, or no changes on symptoms. When doing this, practitioners attempted to crosscheck patients' experiences by using a closed question after providing feedback on total scores: ‘Does that seem accurate?’ (Patient ID 106‐Practitioner ID 26‐Session 1), ‘Does that feel right?’ (Patient ID 022‐Practitioner ID 07‐Session 1), ‘Does that kind of fit with how things have been?’ (Patient ID 100‐Practitioner ID 03‐Session 1), ‘Is that reflective of how you are feeling at the moment?’ (Patient ID 094‐Practitioner ID 04‐Session 1).

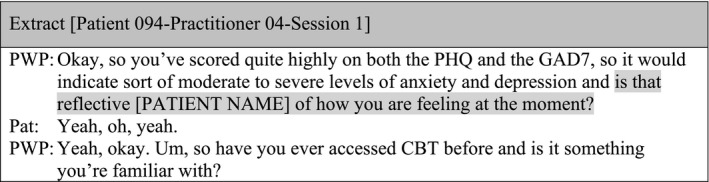

In contrast to the use of closed questions, there was only one example in the data set where the practitioner used an open question in Session 2 inviting the patient to think about what he/she has been doing differently between sessions to understand the reduction in total scores: ‘But what do you think has kind of happened over the last week to kind of have such a big change on your scores?’ (Patient ID 013‐Practitioner ID 06‐Session 2).

The patient initially said he/she did not know why the scores were reduced but then talked about making changes to their sleep routine, eating healthier, relaxing a bit more, consciously making an effort to feel better and talking about their problems. After this conversation, the patient highlighted that reflecting together on the questionnaires was helpful:

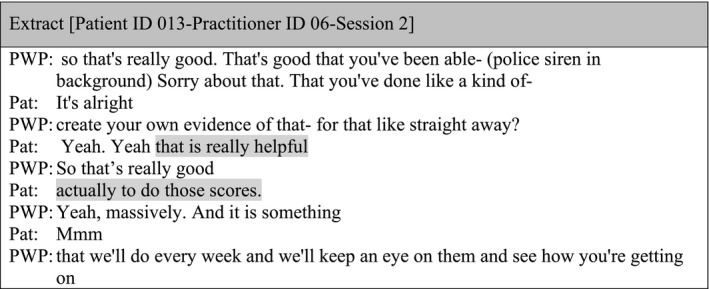

### Theme 4: Missed opportunities to use outcome measures therapeutically

There was a lack of integration and therapeutic use between the administration of outcome measures and decision‐making during treatment sessions. Several examples became apparent through data analysis as missed opportunities to use scores in a therapeutic way. These missed opportunities included the lack of use of (i) item scores and (ii) information gathered from the administration of the IAPT Phobia Scale and the WSAS. This theme comprised two sub‐themes (see Table 2).

#### Sub‐theme 1: Lack of therapeutic use of item scores

Patients' responses to the outcome measures were not explored by the practitioner, missing an opportunity to capture reflections and personalise meaning behind the assigned item scores. In addition, changes in item scores across sessions were usually not discussed. The following extract illustrates information provided by the patient about medication and their perception of its impact on sleeping patterns, but this was not further explored by the practitioner.

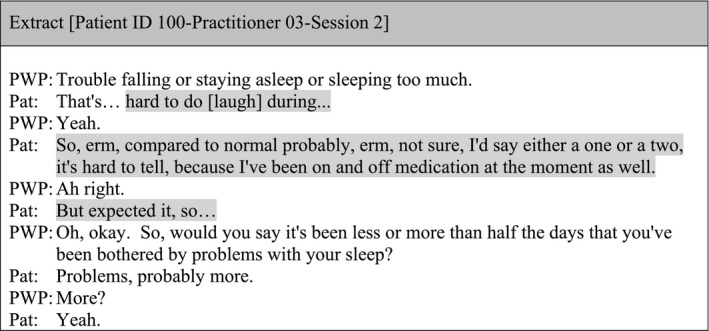

It is important to highlight that all the items included in the PHQ‐9 ask about multiple aspects within an item, e.g., Item 2: feeling: (a) down (b) depressed or (c) hopeless. Similarly, in the GAD‐7, there are three items asking about different facets within an item (Item 1, 2 and 6), for example Item 1: Feeling (a) nervous, (b) anxious or (c) on edge. Questions exploring how the patient is rating items including multiple aspects would help to understand responses and inform treatment decisions.

**FIGURE 1:**
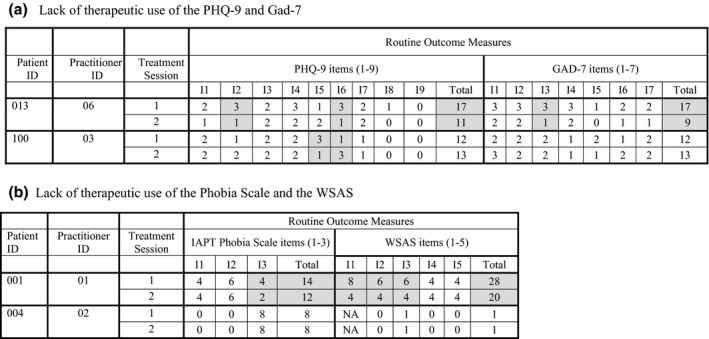
Lack of therapeutic use of item and total scores. (a) Lack of therapeutic use of the PHQ‐9 and gad‐7, (b) lack of therapeutic use of the phobia scale and the WSAS. *Note*: Item‐score information was extracted from treatment session transcripts. Highlights on grey colour indicate a change of two points between treatment sessions. NA = not applicable (i.e. patient was not working)

Focusing on item scores could be particularly relevant to inform the session when total scores show a deterioration or no changes across sessions, a careful look into item scores could help to identify changes that might get lost when attention is on the total score. Figure 1a includes an example of two patients highlighting changes in items by two points between sessions that could be used to facilitate a conversation to explore reasons behind fluctuations in scores.

#### Sub‐theme 2: Lack of therapeutic use of the Phobia Scale and the WSAS

The lack of therapeutic use of outcome measures extends to the Phobia Scale and the WSAS, which were administered in the majority of the sessions (i.e. 11 sessions). However, these data were not utilised during the session and further details were not gathered to personalise scores.

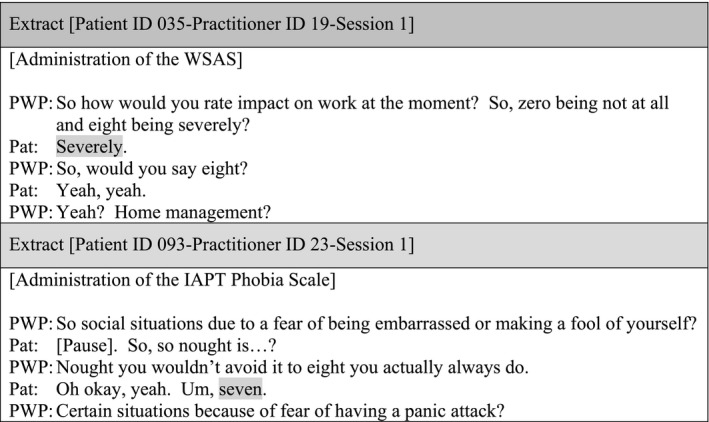

The PHQ‐9 and/or the GAD‐7 may not capture the key issue of importance, and therefore tracking changes on these additional measures could be helpful to monitor patient progress and prevent an improvement in depression and/or anxiety measures mislead treatment outcomes. If an improvement in the PHQ‐9 and/or GAD‐7 is not accompanied by better functioning in life (e.g. WSAS), this should be addressed before the end of treatment to improve the likelihood of long‐term recovery**.** Figure 1b shows an example of two patients highlighting changes in items by two points across sessions or no changes.

## DISCUSSION

This study explored the administration and therapeutic use of session‐by‐session routine outcome measure within and between treatment sessions in psychological interventions delivered by telephone at Step 2 care in IAPT services. Findings indicated a lack of consistency in the administration of the outcome measures within and across practitioners, posing a question about the validity, reliability and meaningfulness of the data collected during telephone‐treatment sessions. Therefore, implementation procedures should be in place to ensure high‐quality data (e.g. use exact wording when referring to items). During COVID‐19, many services asked patients to complete measures online prior to the session; these implementation procedures are still valid when practitioners refer back to items.

Findings highlight the lack of clinical use of outcome measures within and between treatment sessions. This could be explained by previous studies into the attitudes of mental health practitioners on outcome measures which highlighted practitioners did not see the value of using outcome measures as they felt they were capable of assessing and monitoring their own work (Hatfield & Ogles, 2004). In addition, in time‐limited sessions (30 min in Step 2 care), PWPs reported concerns about the proportion of time and effort spent completing measures (Faija et al., 2020). The administration of the outcome measures was perceived as a bureaucratic exercise instead of a clinically meaningful tool (Happell, 2008). This is in line with patients' thoughts about measures being taken to monitor service effectiveness (Solstad et al., 2019).

Findings from the current study reflect the challenges encountered in routine practice towards the understanding and implementation of routine outcome measures; attention to its clinical use may continue to improve IAPT service delivery. The research study points to the need to enhance the therapeutic yield from session‐by‐session outcome measures focusing on three main areas: adopting a collaborative conversational approach, maximising the use of total and items scores, and integrating outcome measures within‐session treatment decisions.

In terms of adopting a *collaborative conversational approach*, this requires PWPs in their training to be made aware of the value of embracing skills such as Psychodynamic Interpersonal Empathy (PI‐E; Guthrie et al., 2018), an empathy skills training package specifically designed for PWPs in the IAPT programme. In essence, the programme focuses on skills to enhance the bond between patient and PWP, as well as the ability of the therapist to create positive expectations through an explanation and understanding of the person's problems. Hence the collection of information on items and measures is construed as a conversation in which the patient is able to be more engaged in the process, what may improve their qualitative experience and facilitate the development of a good therapeutic alliance. PI‐E training has been found to reduce non‐attendance rates (Taylor, 2017), a finding that mirrors a meta‐analysis investigating the effect of providing feedback on outcome measures which yielded a reduction of 20% in patient dropout rates (de Jong et al., 2021). Implementation of PI‐E following deliberate practice (Ericsson et al., 1993; Rousmaniere, 2017) may help to focus on developing practitioner skills in responding with empathy to patient‐reported items and measures.

Within such a conversational empathic frame, there is a need to *maximise the use of total and item scores* from all outcome measures by understanding and approaching them as *clinical process tools* rather than an administrative task to fulfil service requirements. This process can facilitate capturing the meaning behind the item numbers, thereby better enabling both PWP and patient to understand the nature of a patient's problems, symptoms and experiences, and the contextual circumstances that may explain the maintenance of psychological distress and the impact of the problem in patients' functioning. Examples including how to maximise the use of scores may involve facilitating a conversation on improvements at item and total score levels to instil hope and positive praise for the patient. Focusing on deterioration in scores could be an opportunity to think about specific events, thoughts and behaviours that took place between sessions; while fluctuations or no changes in scores could be used to normalise up and downs or reflect on stability and emphasise the importance on the treatment journey as the bigger picture. Empirical and clinical value of session‐by‐session monitoring using feedback is well documented in the literature (e.g. de Jong et al., 2021; Delgadillo et al., 2018; Lambert et al., 2018; Solstad et al., 2019), including tracking responses at an item level (Cross et al., 2015) and thereby promoting a conversational approach towards the administration of the outcome measures. Increasing practitioners' knowledge on the evidence and clinical benefits of outcome measures may help to change underlying negative beliefs/attitudes, what should be explicitly addressed during training and supervision.

Adopting a conversational style together with viewing individual item changes as clinical process tools leads to *integrating outcome measures at a granular level in the therapeutic process*. As such, practitioners move from administering outcome measures as a stand‐alone exercise aiding discharge, to one of focusing on item changes and their potential as a constituent part of the therapeutic encounter to embed measures in treatment and enhance the process of clinical practice. Sensitive and meaningful integration of feedback in therapeutic practice can be conceptualised as a clinical skill, which should be part of training programmes for mental health practitioners; and following‐up this ability within clinical supervision could prove beneficial. This is in line with the CBT competence framework developed by Roth and Pilling (2007) which is included in the IAPT Manual (Version 5, 2021). Further emphasis on how to develop and implement this ability in practice should be placed on the Richards and Whyte (2011) materials, usually used to support the delivery of training for low intensity interventions in IAPT. Introducing the measures as a therapeutic tool may enhance engagement from patient and practitioner towards its use. We are mindful that such processes place greater demands on a PWP's time constraints. However, we encourage approaching measures as clinical process tools by adhering to their completion while facilitating a dialogue to promote personalised care provision.

### Strengths and limitations

The use of data collected from practitioners under usual working conditions provides a context in which to advance our understanding of the administration and therapeutic use of outcome measures during telephone treatment. However, sessions were recorded, meaning practitioners were aware that transcripts would be analysed and this may have influenced the way the sessions were delivered. It could be argued that practitioners who felt most comfortable delivering treatment by phone might have self‐selected. We have used thematic analysis to analyse our data; however, other alternative qualitative approaches, such as framework analysis, could prove fruitful to manage larger data sets among multi‐disciplinary health teams (Gale et al., 2013); where the use of a framework/matrix will facilitate the process of analysis.

The study had a number of limitations. Data were limited to treatment sessions 1 and 2 and not all practitioners across the services consented to participate: of the 18 practitioners who did consent, 11 provided recordings of treatment sessions and only five corresponded to the same practitioner‐patient dyad. However, data analysis identified differences and similarities in the use of session‐by‐session measures within sessions 1 and 2 across practitioners.

Future research should explore patient and practitioner experiences of approaching outcome measures as clinical process tools (i.e. adopting a collaborative, conversational and empathic style), and its potential impact on dropout rates and on clinical outcomes. Furthermore, research investigating administration and clinical use of outcome measures by telephone at Step 3 could enlighten similarities/differences compared to Step 2 care.

## CONCLUSION

A collaborative conversational approach towards the administration of session‐by‐session measurement and the clinical use of item and total scores to inform the treatment work, offer a valuable strategy for tailoring and personalising patient progress that is aligned with the values of patient‐centred care (Department of Health, 2000). This more granular approach has the potential to aid specificity and personal meaning from a patient's perspective. Shifting the perception of outcome measures from impersonal numbers to being clinical tools has the potential to enhance engagement from practitioners and patients, which may result in a better qualitative experience, and which may potentially reduce early attrition rates and improve clinical outcomes. We would recommend placing more emphasis/dedicated time to the ability to use outcome measures from a collaborative conversational approach; making sure this is within the low intensity competencies and is consequently assessed during training and monitored in clinical supervision.

## AUTHOR CONTRIBUTIONS

**Cintia L. Faija:** Conceptualization; Formal analysis; methodology; Writing – original draft; Writing – review & editing. **Penny Bee:** Funding acquisition; Writing – review & editing. **Karina Lovell:** Funding acquisition; Writing – review & editing. **Nicky Lidbetter:** Funding acquisition; Writing – review & editing. **Judith Gellatly:** Conceptualization; Project administration; Supervision; Writing – review & editing. **Kerry Ardern:** Conceptualization; Writing – review & editing. **Kelly Rushton:** Conceptualization; Writing – review & editing. **Helen Brooks:** Conceptualization; Funding acquisition; Writing – review & editing. **Dean McMillan:** Funding acquisition; Writing – review & editing. **Christopher J. Armitage:** Funding acquisition; Writing – review & editing. **Rebecca Woodhouse:** Writing – review & editing. **Michael Barkham:** Conceptualization; Funding acquisition; methodology; Supervision; Writing – original draft; Writing – review & editing.

## CONFLICTS OF INTEREST

The authors declare they have no competing interests.

## Data Availability

The data set generated and analysed during the current study is not publicly available due to privacy and ethical restrictions (i.e. potential for breach of anonymity) but is available from the corresponding author upon reasonable request.
